# Glutamate Cotransmission in Cholinergic, GABAergic and Monoamine Systems: Contrasts and Commonalities

**DOI:** 10.3389/fncir.2018.00113

**Published:** 2018-12-18

**Authors:** Louis-Eric Trudeau, Salah El Mestikawy

**Affiliations:** ^1^CNS Research Group, Department of Pharmacology and Physiology, Department of Neurosciences, Faculty of Medicine, Université de Montréal, Montreal, QC, Canada; ^2^Department of Psychiatry, Faculty of Medicine, Douglas Mental Health University Institute, McGill University, Montreal, QC, Canada; ^3^Sorbonne Universités, Université Pierre et Marie Curie UM 119—CNRS UMR 8246—INSERM U1130, Neurosciences Paris Seine—Institut de Biologie Paris Seine (NPS—IBPS), Paris, France

**Keywords:** cotransmission, glutamate, vesicular transporters, synapse, VGLUT

## Abstract

Multiple discoveries made since the identification of vesicular glutamate transporters (VGLUTs) two decades ago revealed that many neuronal populations in the brain use glutamate in addition to their “primary” neurotransmitter. Such a mode of cotransmission has been detected in dopamine (DA), acetylcholine (ACh), serotonin (5-HT), norepinephrine (NE) and surprisingly even in GABA neurons. Interestingly, work performed by multiple groups during the past decade suggests that the use of glutamate as a cotransmitter takes different forms in these different populations of neurons. In the present review, we will provide an overview of glutamate cotransmission in these different classes of neurons, highlighting puzzling differences in: (1) the proportion of such neurons expressing a VGLUT in different brain regions and at different stages of development; (2) the sub-cellular localization of the VGLUT; (3) the localization of the VGLUT in relation to the neurons’ other vesicular transporter; and (4) the functional role of glutamate cotransmission.

## Introduction on Cotransmission and the Discovery of VGLUTs

**Figure 1:**
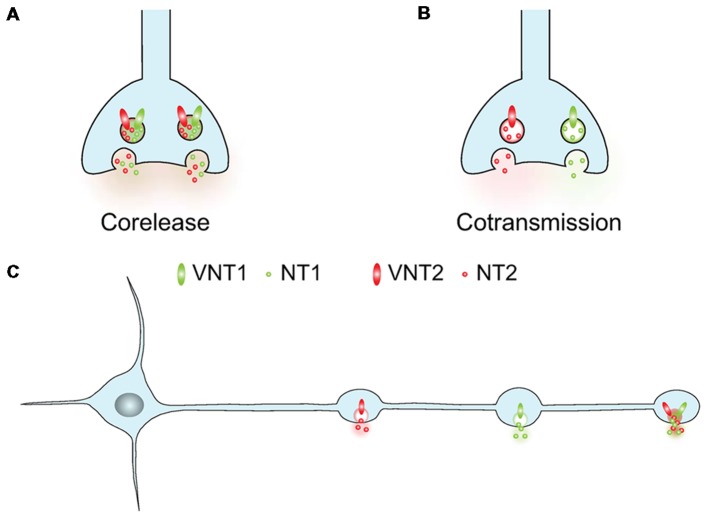
The co-expression of two vesicular neurotransmitter transporters by a single neuron generates multiple signaling possibilities. **(A,B)** Two different vesicular neurotransmitter transporters (VNT1 or VNT2) can either be addressed to the same synaptic vesicles (SVs) or segregated in different SVs. In the first case the two transmitters (NT1 and NT2) will be coreleased simultaneously, in the second one they can be differentially released. **(C)** Within a neuron, two different VNTs can be targeted the same or to different varicosities. With these two options, a neuron can release two different NTs from various varicosities or corelease two NTs from a single varicosity.

*Stricto sensu*, cotransmission implies that two neurotransmitters are released from the same neuron. The complementary term “corelease” is considered by many to imply simultaneous release of two transmitters from the same vesicles. For corelease to occur, two classic transmitters [glutamate, GABA, dopamine (DA), acetylcholine (ACh), serotonin (5-HT), norepinephrine (NE)] must be stored within the same synaptic vesicle (SV) in the readily releasable pool (RRP). In the absence of actual corelease from the same SVs (Figure 1A), cotransmission could involve the synchronous release of two different sets of vesicles containing different neurotransmitters (Figure 1B). In this review we will use the term cotransmission in a broad way to refer to neurons that can release more than one classic transmitter (glutamate, GABA, DA, ACh, 5-HT, NE). This release sometimes occurs from the same terminals, but it could also arise from different varicosities established by a given neuron (Figure 1C).

Glutamate is the major excitatory transmitter in the brain. To transmit an excitatory signal, neurons must have the capacity to accumulate glutamate inside SVs that will undergo regulated fusion with the synaptic plasma membrane (Takamori et al., 2000, 2001). Proton-dependent solute carriers fulfill this critical accumulation of glutamate in SVs. These transporters belong to the SLC17 family and include VGLUT1 (SLC17A7), VGLUT2 (SLC17A6) and VGLUT3 (SLC17A8; for review, see El Mestikawy et al., 2011; Anne and Gasnier, 2014). VGLUT1-3 are secondary transporters and their activity is driven by a proton gradient established by the vacuolar-type H^+^-ATPase (V-ATPase; Edwards, 2007; Omote et al., 2011). VGLUT1 was the first member of the family to be identified and was initially characterized as a putative brain-specific Na^+^-dependent transporter of inorganic phosphate (Pi; originally named BNPI; Ni et al., 1994). The H^+^-driven transport of glutamate inside SVs by VGLUT1 was demonstrated 6 years later (Bellocchio et al., 2000; Takamori et al., 2000). Interestingly, heterologous expression of “BNPI” in GABA primary neurons was found to be sufficient to confer a glutamatergic phenotype to these inhibitory neurons (Takamori et al., 2000), demonstrating that vesicular glutamate transporters (VGLUTs) are key to the acquisition of a glutamatergic phenotype by neurons. Taken together, these initial studies suggested that VGLUTs were able to transport two different substrates (glutamate and/or Pi) in different cellular locations (plasma membrane or SVs) and in opposite directions. This inversely oriented transport of glutamate and Pi remained controversial for more than two decades. However, a recent publication solved this mystery and established that VGLUTs are indeed dual carriers. Preobraschenski et al. (2014) recently demonstrated elegantly that VGLUT1 co-accumulates glutamate and Pi inside SVs in a proton-dependent manner when it is facing the cytoplasm. However, when facing the extracellular space, VGLUT1 mediates cytoplasmic Pi accumulation in a Na^+^-dependent manner (Preobraschenski et al., 2014). In addition to glutamate and Pi, VGLUT1 potentially also transports chloride ions (Naito and Ueda, 1985; Maycox et al., 1988; Takamori, 2016; Preobraschenski et al., 2014). The influx of Pi in the cytoplasm of glutamatergic terminals could help to activate Pi-activated glutaminase and hence to replenish glutamate stores (Masson et al., 2006). However, the rationale for the transport of glutamate, Pi and chloride by VGLUT1 is not fully understood and remains to be formally established for VGLUT2 and VGLUT3.

Similarly to VGLUT1, VGLUT2 was first categorized as a Pi transporter and named DNPI (Aihara et al., 2000), shortly before its ability to accumulate glutamate in SVs was established by several teams (Bai et al., 2001; Fremeau et al., 2001; Herzog et al., 2001; Takamori et al., 2001; Varoqui et al., 2002). VGLUT1 and VGLUT2 are very similar in terms of structure and glutamate transport. However, they differ mainly by their distribution and by the probability of glutamate release they confer to neurons expressing them (Herzog et al., 2001; Fremeau et al., 2004; Herman et al., 2014).

In 2002, VGLUT3 was the last subtype of vesicular glutamate transporter to be identified (Fremeau et al., 2002; Gras et al., 2002; Schäfer et al., 2002; Takamori et al., 2002). This delayed discovery probably reflects the low abundance of VGLUT3 compared to VGLUT1 or VGLUT2. Despite its functional and structural similarity with VGLUT1 and VGLUT2, VGLUT3 displays some atypical anatomical features. For example, VGLUT3 is found in a small population of glutamatergic neurons present in raphe nuclei, striatum or cortex and in inner hair cells. In addition, VGLUT3 is expressed by subpopulations of neurons releasing 5-HT, GABA (CCK-positive basket cells) or ACh (for review see El Mestikawy et al., 2011). VGLUT3 is also transiently expressed by GABA neurons of the cerebellum and auditory system (Gillespie et al., 2005; Gras et al., 2005; Case and Gillespie, 2011; Case et al., 2014). As will be further discussed below, VGLUT1 and VGLUT2 were also reported in subpopulations of ACh, DA or GABA neurons, although this expression can be dependent of the developmental stages (Dal Bo et al., 2004; Trudeau, 2004; Mendez et al., 2008; Zander et al., 2010; Ren et al., 2011). Electrophysiological and optogenetic methods played a decisive role to establish that in numerous cases, glutamate was indeed released by 5-HT neurons (Johnson, 1994; Varga et al., 2009; Sengupta et al., 2017), DA neurons (Sulzer et al., 1998; Bourque and Trudeau, 2000; Hnasko et al., 2010; Tecuapetla et al., 2010; Chuhma et al., 2014; Trudeau et al., 2014), ACh (Huh et al., 2008; Higley et al., 2011; Nelson et al., 2014; Frahm et al., 2015) and GABA neurons (Shabel et al., 2014; Root et al., 2018).

In summary, VGLUT1-3 are observed in numerous neuronal populations that were initially not considered glutamatergic. An implication of these discoveries is that glutamate cotransmission must regulate numerous brain functions. This issue will be discussed in section “What Are the Functional Implications of Glutamate Cotransmission in These Neuronal Populations?”. But to date, optogenetic experiments convincingly established that glutamate cotransmission regulates reward-related behaviors (Birgner et al., 2010; Hnasko et al., 2010; Witten et al., 2010; Adamantidis et al., 2011; Alsiö et al., 2011; Liu et al., 2014) and auditory or respiratory functions (Abbott et al., 2014; Burke et al., 2014). The implication of glutamate cotransmission in other brain functions such as memory, fear or stress is not clearly established (Balázsfi et al., 2018).

## Characterization of VGLUTs in Monoamine, Cholinergic and GABA Neurons Reveals Highly Heterogeneous Expression in Different Neuronal Populations

The identification of VGLUTs in monoamine, cholinergic and GABA neurons raised a lot of interest in the potential roles of glutamate released as an additional neurotransmitter by these neurons. Exploration of the pattern of expression of these transporters in such neurons, mostly in the adult brain, revealed surprises. One of these is that in these different neuronal populations, there are wide variations in the proportion of neurons containing detectable levels of a VGLUT in the adult rodent brain. As an example, in the DA system, while subsets of ventral tegmental area (VTA) DA neurons contain VGLUT2 and release glutamate in the ventral striatum, adult *substantia nigra* *compacta* (SNc) DA neurons typically contain only very low levels or no vglut2 mRNA and thus do not establish many glutamatergic synapses in the dorsal striatum, as revealed by optogenetic stimulation and patch-clamp electrophysiology (Stuber et al., 2010). The examination of the distribution of vglut2 mRNA in the DA system revealed the existence of higher proportions of DA neurons containing vglut2 mRNA in the rostro-medial regions of the VTA (Yamaguchi et al., 2007; Li et al., 2013). The exact proportion of DA neurons containing vglut2 mRNA was found to be very low in some studies using *in situ* hybridization; approximately 0.1% of TH-positive neurons in the VTA were found to contain vglut2 mRNA in one of these studies performed in rats (Yamaguchi et al., 2007). In a separate study of the SNc, again in rats, these same authors reported similar numbers, with slightly less than 0.1% of SNc TH-positive neurons containing vglut2 mRNA (Yamaguchi et al., 2013). But such low proportions are likely to result from technical limitations as other studies reported much higher proportions in the VTA (30%–50%), both using* in situ* hybridization (Li et al., 2013) and single-cell RT-PCR (Mendez et al., 2008; Fortin et al., 2012; Li et al., 2013). Although much less studied at early developmental stages, the glutamatergic phenotype of DA neurons appears to be the rule rather than the exception early in development; estimates suggest that over 80% of DA neurons in the VTA and SNc expressed the vglut2 gene at some point of their embryonic development (Dal Bo et al., 2008; Steinkellner et al., 2018). Similarly to DA neurons, only a subset of NE/epinephrine neurons appears to display a glutamatergic phenotype and express vglut2. But in this system there is a striking distinction between different subgroups, with the majority (>80%) of C1, C2 and C3 adrenergic neurons and A2 NE neurons expressing vglut2 (Stornetta et al., 2002a,b; DePuy et al., 2013), partial (16%) expression in the A1 group and essentially no expression in the locus coeruleus (LC; Stornetta et al., 2002a). In keeping with these observations, epinephrine and NE neurons establish VGLUT2-positive synapses in target areas including for example the spinal cord and the dorsal motor nucleus of the vagus (Nakamura et al., 2004; DePuy et al., 2013). 5-HT neurons of the raphe nuclei are comparatively less heterogeneous in their expression of VGLUT3. Although initial studies proposed that most 5-HT neurons of the dorsal and medial raphe expressed *vglut3* mRNA (Gras et al., 2002; Herzog et al., 2004), subsequent investigations concluded that in fact only approximately 50% do so (Hioki et al., 2010; Voisin et al., 2016; Sos et al., 2017).

Cholinergic neurons in the brain are also dichotomous in their expression of VGLUT3. On one hand, most if not all cholinergic interneurons of the striatum express VGLUT3 (Gras et al., 2002; Herzog et al., 2004). On the other hand, within the basal forebrain, the expression of VGLUT3 is highly heterogeneous in cholinergic neurons; in this area approximately 30% of cholinergic neurons in the ventral pallidum of rats contain vglut3 mRNA, while virtually no expression is detected in the medial septum and vertical limb of the diagonal band of Broca (Nickerson Poulin et al., 2006). However, using a genetic fate-mapping approach, it was reported that approximately 50% of cholinergic neurons of the horizontal limb of the diagonal band of Broca in mice have a history of vglut3 expression (Case et al., 2017).

The expression of VGLUTs in GABA neurons also illustrates broad heterogeneity. VGLUT3 is present in GABAergic basket cells of the hippocampus and cortex (Fremeau et al., 2002; Gras et al., 2002; Hioki et al., 2004; Somogyi et al., 2004; Fasano et al., 2017). In the CA1 and CA3 regions, it is only present in approximately 10%–25% of CCK-positive interneurons, with no expression in VIP-positive interneurons (Somogyi et al., 2004; Fasano et al., 2017; Del Pino et al., 2017). Similarly, in the basal amygdala, vglut3 mRNA is found in approximately 25% of CB1/CCK-positive GABAergic neurons (Omiya et al., 2015). In the bed nucleus of the stria terminals, only less than 10% of GABA neurons appear to express vglut3 (Kudo et al., 2012). In a recent study, neurons containing both VGLUT2 and the GABAergic markers glutamic acid decarboxylase (GAD) and vesicular GABA transporter VGAT were detected in the VTA, entopeduncular nucleus (EPN) and supramammillary (SUM) nucleus (Root et al., 2018). In the VTA, a little over 20% of neurons were found to have this phenotype, while in the entopeduncular and lateral supramammillary nuclei, this was approximately 50% and 35%, respectively.

Together, these findings clearly show that VGLUT expression is highly heterogeneous in multiple classes of neurons, suggesting the possibility that cotransmission may allow sub-circuits to be defined within existing projections.

## Studies of the Subcellular Localization of VGLUTs Reveal Complex Modes of Cotransmission

The complexity of glutamate cotransmission is further highlighted in studies that examined the subcellular localization of VGLUTs in neurons using glutamate as a cotransmitter. Indeed, whether VGLUTs in neurons using other neurotransmitters are localized to the same terminals releasing the neuron’s other classical transmitter, or even on the same vesicles, places some clear constraints on the mode of cotransmission. In DA neurons, initial studies evaluating the localization of glutamate immunoreactivity in single neuron cultures highlighted the existence of terminals positive for glutamate but negative for the DA biosynthetic enzyme tyrosine hydroxylase (TH; Sulzer et al., 1998). This suggested that release sites for glutamate and DA might be at least partially segregated along axonal segments (Figure 1). Subsequently, following the immuno-purification of SVs from rat ventral striatum, evidence was provided suggesting that at least a subset of vesicles containing VMAT2 co-immuno-precipitated with vesicles containing VGLUT2 (Hnasko et al., 2010). This suggested that, at least in juvenile rats, a subset of vesicles may contain both vesicular transporters and hence transmitters. However, subsequent anatomical investigations did not support an extensive colocalization of glutamatergic and dopaminergic markers in axon terminals established by DA neurons in both adult rats and mice. First, immuno-localization of VMAT2 and VGLUT2 at the ultrastructural level in rat nucleus accumbens revealed that although the two proteins could be detected in closely located terminals along a given axon, they are essentially never colocalized (Zhang et al., 2015). A similar conclusion was reached in mouse nucleus accumbens for the localization of VGLUT2 and dopaminergic markers like TH and DAT in two independent studies (Zhang et al., 2015; Fortin et al., 2018). Together these results suggest that if VGLUT2 and VMAT2 can possibly be found on the same vesicles in a subset of vesicles or at early developmental periods, DA and glutamate release sites established along dopaminergic axons appear to be mostly segregated.

A similar segregation of 5-HT terminal markers and VGLUT3 in the axonal projections of raphe neurons in the mouse has also been reported both *in vitro*, in single neuron cultures (Fremeau et al., 2002) and *in vivo* (Schäfer et al., 2002; Nakamura et al., 2004; Voisin et al., 2016). Only a subset of VGLUT3-positive terminals established by 5-HT neurons in regions including the striatum, the lateral septum, the hippocampus and the spinal cord are immuno-positive for the 5-HT reuptake site SERT or 5-HT itself. Co-immunostaining for VMAT2 and VGLUT3 also provided evidence for heterogeneous sets of terminals in the cortex and hippocampus, with only a subset expressing both markers (Schäfer et al., 2002; Amilhon et al., 2010). Surprisingly, in contrast to these results, one study performed in the rat suggested that most SERT-positive terminals established by individually-labeled dorsal raphe neurons are also VGLUT3-positive (Gagnon and Parent, 2014). The segregation of VGLUTs in the terminals of DA and 5-HT neurons may possibly find its origin in the differential trafficking of VMAT2 and VGLUTs, as suggested in an* in vitro* study studying the trafficking of VMAT2 and VGLUT1 in hippocampal and dopaminergic neurons (Onoa et al., 2010).

The situation appears to be drastically different in adrenergic neurons as, for example, most terminals of adrenergic C1 neurons in the dorsal motor nucleus of the vagus, visualized after conditional expression of ChR2, appear to be TH-immunoreactive and to also be VGLUT2-immunopositive (DePuy et al., 2013). Intriguingly, adrenergic projections in the paraventricular nucleus of the hypothalamus of both rats and mice appear to be more heterogeneous in their expression of VGLUT2, with only approximately 20% of terminals labeled with the adrenaline biosynthetic enzyme phenylethanolamine N-methyltransferase (PNMT) also positive for VGLUT2 (Johnson et al., 2018). Similarly, only a subset of DA beta-hydroxylase-positive terminals in the intermediolateral cell column of the spinal cord were found to be VGLUT2-postive (Nakamura et al., 2004).

In cholinergic interneurons, VGLUT3 is abundantly expressed in axonal-like varicosities containing the vesicular ACh transporter (VAChT) or choline acetyltransferase (ChAT; Gras et al., 2002, 2008; Sakae et al., 2015; Kljakic et al., 2017). Immuno-isolation of cholinergic vesicles from the rat striatum further suggested that VAChT and VGLUT3 may be together on subsets of SVs (Figure 1), a configuration allowing for a synergistic interaction between the two transmitters, leading to enhanced vesicular packaging of ACh or glutamate, a mechanism named vesicular synergy (Gras et al., 2008; Frahm et al., 2015). But an evaluation of cholinergic terminals emanating from basal forebrain cholinergic neurons revealed that while VGLUT3 and ChAT are highly co-localized in terminals in regions such as the basolateral amygdaloid nucleus, they are typically found on separate populations of terminals in other regions including the olfactory bulb, the reticular thalamic nucleus and the entorhinal cortex (Nickerson Poulin et al., 2006). VGLUT3 is also found in subsets of GABAergic terminals. For example, VGLUT3 is transiently found with the vesicular GABA/glycine transporter (VIAAT or VGAT) in the axon terminals of neurons of the medial nucleus of the trapezoid body in the lateral superior olive (Gillespie et al., 2005). In these projections, it appears that only a subset of terminals labeled with VIAAT are also immuno-positive for VGLUT3 (Gillespie et al., 2005). A subset of GABAergic terminals co-labeled with VGLUT3 has also been reported in the intermediolateral cell column of the spinal cord (Stornetta et al., 2005). In the terminals of hippocampal GABA neurons, ultrastructural and biochemical evidence for the presence of VGLUT3 on vesicles containing VIAAT has been provided (Stensrud et al., 2013, 2015). Indeed, VGLUT3 seems to be expressed by VIAAT-positive SVs and furthermore these SVs are able to accumulate [^3^H]glutamate (Fasano et al., 2017). Arguing in favor of the fact that such colocalization could lead to corelease of glutamate and GABA from the same vesicles (Figure 1), overexpression of VGLUT3 in GABA neurons *in vitro* has been shown to allow for co-occurring GABA- and glutamate-mediated synaptic currents (Zimmermann et al., 2015).

In contrast to these previous results highlighting release of GABA and glutamate from the same terminals, recent work characterizing the co-expression of VGLUT2, VIAAT and glutamic acid decarboxylase (GAD) by neurons of the VTA, EPN and SUM nuclei (Root et al., 2018), provided evidence for segregation of release sites for the two neurotransmitters. These authors reported that GABA/glutamate neurons from the VTA or EPN send projections to the lateral habenula (LHb) where they form asymmetric (excitatory) and symmetric (inhibitory) synapses. However, these “mixed” axons segregate VGLUT2 and VIAAT onto separate SVs and therefore appear to release GABA or glutamate from distinct release sites, further highlighting the complex pattern adopted by various co-transmitting neurons (Figure 1).

The presence of VGLUT3 in the somatodendritic compartment of 5-HT, cholinergic and cortical neurons is also particularly intriguing and unique in comparison to VGLUT1 and VGLUT2, that are exclusively present in axon terminals (Fremeau et al., 2002; Gras et al., 2002; Harkany et al., 2004; Herzog et al., 2004; Somogyi et al., 2004; Calizo et al., 2011). It has been considered that VGLUT3-containing vesicles in the dendrites of neurons may be involved in the dendritic release of glutamate and retrograde signaling. This possibility has recently received direct support from experiments showing that stimulation of the dendrites of glycinergic amacrine cells of the retina leads to VGLUT3-dependent glutamate-mediated activation of ganglion cells (Haverkamp and Wässle, 2004; Johnson et al., 2004; Marshak et al., 2015; Tien et al., 2016; Chen et al., 2017).

## What Are the Functional Implications of Glutamate Cotransmission in These Neuronal Populations?

Despite the widespread existence of glutamate cotransmission in the brain and much recent progress, we still know very little about its molecular and cellular modalities or its functional implications. A question that arises is whether two neurotransmitters within the same synapse influence each other’s signaling? The existence of vesicular synergy clearly illustrates how glutamate, perhaps through its negative charge, can influence the quantum size of ACh-, DA-, 5-HT- and GABA-containing SVs and conversely, how ACh can increase the accumulation of glutamate in SVs (Gras et al., 2008; Amilhon et al., 2010; Hnasko et al., 2010; Zander et al., 2010; Frahm et al., 2015; Voisin et al., 2016). Interestingly, in heterologous cells (HEK293T) cotransfected with VGLUT2 and VMAT2, glutamate (through the presence of VGLUT2) produces a robust and stable acidification of SVs (Hnasko et al., 2010). The activity of VMAT2 and VAChT is strongly dependent on the existence of the intralumenal ΔpH. Therefore, the basic mechanism underlying vesicular synergy could be the existence of this increased influx of protons due to VGLUT2 or VGLUT3 activity. The existence of such a mechanism has received partial support from functional experiments carried out in drosophila DA neurons. Aguilar et al. (2017) reported that neuronal depolarization induces a VGLUT-dependent hyper acidification of SVs that causes a small increase in the vesicular uptake of FFN206, a fluorescent VMAT substrate. These data provide some support for the hypothesis that the vesicular quantum of neurotransmitter is modulated at least in part by neuronal activity and that vesicular synergy could help to dynamically increase DA content in SVs to meet the varying demands of neuronal activity. However, for such vesicular synergy to occur, both vesicular transporters need to be present on at least a subset of the same SVs. However, the evidence in favor of the co-expression of VGLUT2 and VMAT2 on the same vesicles is not strong. While immunopurification of SVs from juvenile rat striatum suggested co-purification of vesicles containing both vesicular transporters (Hnasko et al., 2010), others provided evidence for differential localization of both transporters to different axon terminals in the mouse (Zhang et al., 2015; Fortin et al., 2018). In the drosophila brain, it has been estimated that less than 5% of dopaminergic terminals contain the drosophila VGLUT (Aguilar et al., 2017).

The presence of VAChT and VGLUT3 on the same vesicles received support from functional experiments (effect of glutamate on ACh vesicular accumulation i.e., vesicular synergy) and by vesicle immuno-purification experiments (Gras et al., 2008). However, these are bulk methods that could lead to “false-positive” vesicular colocalization. For this and other suspected cases of vesicular synergy, higher resolution imaging techniques such as Stimulated Emission Depletion (or STED) microscopy (Hell and Wichmann, 1994) or immuno-electron microscopy are needed to provide stronger support for co-localization at the level of single vesicles. STED has recently been used to investigate the distribution of VGLUT3 variants in cholinergic varicosities (Ramet et al., 2017) and could help to examine the presence of VGLUTs and other vesicular transporters on the same SVs.

Recently, in SV immunopurification experiments, VGLUT2 and VGAT were also found to be present in different sets of vesicles in the rat LHb, an observation confirmed by immuno-gold electron microscopy (Root et al., 2018).

Broadly speaking, glutamate corelease may act to amplify postsynaptic activation of target cells, either through activation of ionotropic or metabotropic glutamate receptors. In line with this, genetic deletion of VGLUT3 from striatal cholinergic interneurons leads to impaired activation of striatal GABAergic fast-spiking interneurons (Nelson et al., 2014). But glutamate released through cotransmission can also activate metabotropic glutamate receptors. For example, in the hippocampus, Fasano et al. (2017) recently showed that VGLUT3-mediated glutamate release by basket cells tones-down local GABA transmission by stimulating metabotropic glutamate receptors. This inhibition of local GABA tone by VGLUT3-dependent glutamate alters hippocampal network properties (plasticity and oscillations). The presence of VGLUT3 in basket cells could thus have important consequences for spatial learning or mood regulation.

However, we still do not know how and when glutamate and other transmitters are coreleased from VGLUT3-positive terminals. For example, are the two transmitters released simultaneously or differentially? This question has not been tackled yet in the striatum or in the hippocampus. However, interesting data have started to accumulate in other brain areas such as the interpeduncular nucleus (IPN) or the amygdala. In these areas, such “bilingual” neurons release either glutamate or their cognate transmitter (ACh or 5-HT) depending on their firing pattern. The IPN receives a dense cholinergic innervation from the medial Hb; these terminals massively co-express VGLUT1 (Ren et al., 2011). In the IPN, brief optogenetic stimulation of medial Hb terminals produces fast glutamatergic EPSCs, whereas tetanic stimulation is necessary to evoke slower nicotinic responses (Ren et al., 2011). Therefore, quite surprisingly, these so-called “cholinergic” neurons release more easily glutamate than ACh. Interestingly, in these fibers, ACh was shown to synergistically increase glutamate accumulation and release (Frahm et al., 2015), in line with the previous demonstration of vesicular synergy between glutamate and ACh by Gras et al. (2005) in the striatum. Similarly, in the basal amygdala, low frequency (≤1 Hz) optogenetic stimulation of 5-HT fibers evokes glutamate release whereas higher frequencies (10–20 Hz) are required to release 5-HT (Sengupta et al., 2017). These two thought-provoking publications therefore suggest that the preferentially-released transmitter by these cholinergic and serotoninergic fibers is glutamate. Overall, they show that glutamate and its associated transmitters (here ACh or 5-HT) can be used in different firing conditions by the same neurons. These important and challenging findings indirectly imply that the two transmitters are stored in different SVs (as depicted in Figure 1B) and seriously question the initially proposed mechanistics of vesicular synergy. In the initial model of vesicular synergy, glutamate was acting as a counter ion allowing the accumulation of additional protons and hence more ACh (see El Mestikawy et al., 2011). This model implies that VGLUT3 and VAChT were present on the same SVs and is clearly in conflict with the differential release of glutamate (at low firing frequency) and ACh or 5-HT (at higher frequency). Therefore, further investigations will be necessary to fully understand vesicular synergy and the differential release of glutamate and ACh or 5-HT from the same neurons.

What are some of the broader functional implications of co-transmission? In other words, are both transmitters used to fulfill similar or different functions? Optogenetic stimulation does not readily allow to answer this question as it typically triggers release of both transmitters, albeit to possibly different extents according to firing frequency, as discussed previously. In contrast, this problem was investigated in cholinergic interneurons with the use of genetic deletion in mice of either VAChT or VGLUT3. As mentioned previously, cholinergic interneurons from the ventral striatum (or nucleus accumbens, NAc) co-express VAChT and VGLUT3 and consequently signal with both ACh and glutamate (Gras et al., 2008; Guzman et al., 2011; Sakae et al., 2015). The involvement of the NAc in reward-guided behavior and vulnerability to substance use disorders has been abundantly documented since the mid-80s and the seminal experiments of Di Chiara and Imperato (1985) and Imperato and Di Chiara (1986). In addition, immunotoxin-mediated ablation of cholinergic interneurons in the NAc clearly established the involvement of cholinergic interneurons in the sensitivity to the rewarding properties of cocaine (Kaneko et al., 2000; Hikida et al., 2001). However, selective deletion of VAChT from these interneurons and hence silencing of ACh signaling had little effect on the psychostimulant or rewarding properties of cocaine (Guzman et al., 2011). In contrast, the ablation of VGLUT3 recapitulates phenotypes reported with ablation of cholinergic interneurons; VGLUT3-knockout (KO) mice were more sensitive to the stimulant and rewarding effects of cocaine (Sakae et al., 2015). Therefore, as described above in the IPN, ACh seemed to play only a modest role and glutamate appeared to be the major transmitter in the regulation of reward-guided behaviors by cholinergic interneurons.

However, ACh and glutamate released by cholinergic interneurons have opposite effects on DA efflux in the NAc. Activation of presynaptic cholinergic receptors (nAChR) located on DAergic fibers powerfully stimulates DA release in the striatum (for review see Exley and Cragg, 2008). Therefore, ACh stimulates DA release in the NAc. In contrast, glutamate released by cholinergic interneurons binds to a metabotropic glutamate receptor (mGLUR) and inhibits DA release (Figure 2 in Sakae et al., 2015). Together with the numerous reports demonstrating that ACh is a key frequency-dependent regulator of DA release in the striatum (Zhou et al., 2001; Rice and Cragg, 2004; Zhang and Sulzer, 2004; Threlfell and Cragg, 2011; Exley et al., 2012; Jennings et al., 2015), these observations suggest that much remains to be learned about the functional roles of ACh release by striatal interneurons.

The conditional ablation of *vglut2* from DA neurons provides further evidence for a key role of glutamate corelease in the regulation of psychostimulant-induced behaviors (Birgner et al., 2010; Hnasko et al., 2010; Alsiö et al., 2011). Selective ablation of *vglut2* from DA neurons blunted locomotor response to cocaine and amphetamine (Birgner et al., 2010; Hnasko et al., 2010). In contrast, and surprisingly, self-administration experiments showed that rewarding properties of cocaine (and sucrose) were enhanced in these mutants mice (Birgner et al., 2010). While initial studies were performed by Cre-driven gene deletion dependent on the DAT, and thus caused deletion of the *vglut2* gene during the embryonic period, a recent study used a tamoxifen-inducible strategy to KO *vglut2* from DA neurons in adult mice. This work reported unaltered locomotor sensitization to amphetamine and cocaine in the absence of VGLUT2 (Papathanou et al., 2018). A possible interpretation of the discrepancy between the effects of embryonic vs. adult KO of *vglut2* on the response of mice to psychostimulants is that embryonic gene deletion leads to perturbed development of the DA system. More work will be needed to examine this question directly. However, some indirect support for this possibility has been obtained. Although constitutive KO of VGLUT2 is lethal immediately after birth due to absence of breathing (Wallén-Mackenzie et al., 2006), conditional KO of this VGLUT in DA neurons during embryonic development using a DAT-Cre driver reduced DA release in the ventral striatum and reduced the density of TH-positive terminals in this brain region (Hnasko et al., 2010; Fortin et al., 2012). Interestingly, as mentioned previously, VGLUT2 appears to be expressed broadly during the embryonic period in mesencephalic DA neurons (Dal Bo et al., 2008; Steinkellner et al., 2018). Glutamate release or other functions of VGLUT2 in these neurons during the embryonic period could therefore play key roles in the early establishment of dopaminergic pathways. Constitutive KO of VGLUT3 in mice has only limited effects on adult brain networks. For example, Voisin et al. (2016) thoroughly examined the effect of deleting VGLUT3 on the maturation of the 5-HT system. Absence of VGLUT3 did not modify the number of 5-HT neurons, nor the global density of their axons or dendrites in the adult brain. However, when examined *in vitro*, VGLUT3-KO 5-HT neurons showed reduced survival, suggesting a potential role in their basal vulnerability.

Clearly much remains to be done to better understand the functional implications of glutamate cotransmission. Further, more extensive studies using conditional and inducible genetic deletion of VGLUTs at different developmental stages in select neuronal populations, coupled with functional analyses will likely provide further insights in the coming years.

In the present review, we provided an overview of glutamate cotransmission in different classes of neurons. What emerges from this global comparison is the particularly heterogeneous nature of glutamate cotransmission in different brain nuclei. There are wide variations in the proportion of neurons expressing a VGLUT in different brain regions and at different stages of development. The sub-cellular localization of VGLUTs in neurons using multiple classical neurotransmitters is also clearly heterogeneous, with frequent segregation of the VGLUTs in relation to the neurons’ other vesicular transporter. Finally, studies of the functional roles of glutamate cotransmission clearly need to be strengthened by additional studies evaluating these roles at different developmental stages and in disease conditions.

## Author Contributions

L-ET and SEM jointly wrote the manuscript.

## Conflict of Interest Statement

The authors declare that the research was conducted in the absence of any commercial or financial relationships that could be construed as a potential conflict of interest.
